# Influence of N1-Methylpseudouridine in Guide RNAs on CRISPR/Cas9 Activity

**DOI:** 10.3390/ijms242317116

**Published:** 2023-12-04

**Authors:** Daria Prokhorova, Anastasiya Matveeva, Alexander Zakabunin, Alexander Ryabchenko, Grigory Stepanov

**Affiliations:** Institute of Chemical Biology and Fundamental Medicine, Siberian Branch of the Russian Academy of Sciences, 630090 Novosibirsk, Russia; dvprohorova1994@mail.ru (D.P.); zakabunin@niboch.nsc.ru (A.Z.);

**Keywords:** CRISPR/Cas9, N1-methylpseudouridine, modifications of guide RNAs

## Abstract

At present, there are many strategies to improve the activity of CRISPR/Cas9. A well-known and effective approach is guide RNA modification. Many chemical guide RNA modifications have been studied, whereas naturally occurring RNA modifications are largely unexplored. N1-methylpseudouridine (m1Ψ) is an RNA base modification widely used in mRNA therapy, and it holds great promise for application in genome editing systems. The present study focuses on investigating the effect of N1-methylpseudouridine on the functioning of CRISPR/Cas9. In vitro cleavage assays helped determine the level of m1Ψ guide RNA modification that is sufficient to cleave the target substrate. By analyzing FAM-labeled dsDNA substrate cleavage, we calculated the kinetic parameters and the specificity scores of modified guide RNAs. Neon transfection and digital PCR enabled us to assess the activity of modified guide RNAs in mammalian cells. Our study shows that the presence of m1Ψ in guide RNAs can help preserve on-target genome editing while significantly reducing the off-target effects of CRISPR/Cas9 in vitro. We also demonstrate that Cas9 complexes with guide RNAs containing m1Ψ allow for genome editing in human cells. Thus, the incorporation of m1Ψ into guide RNAs supports CRISPR/Cas9 activity both in vitro and in cells.

## 1. Introduction

At present, the clustered regularly interspaced short palindromic repeats (CRISPR)-CRISPR-associated protein 9 (Cas9) system has been widely adopted as a main genome editing tool. It has found its applications in fundamental biological research, diagnostics, and therapy [1,2]. The main components of this system are DNA endonuclease Cas9, a short mature CRISPR RNA (crRNA) with a spacer sequence complementary to the DNA substrate, and a trans-activating crRNA (tracrRNA) complementary to the crRNA; alternatively, it can include a combination of crRNA and tracrRNA, i.e., a chimeric single-guide RNA (sgRNA) [3,4]. To recognize the target DNA, CRISPR/Cas9 requires a short conserved protospacer-adjacent motif (PAM, or NGG in the case of *Streptococcus pyogenes* Cas9) [5]. In addition, 10–12 PAM-proximal nucleotides are the seed region of the guide RNA (gRNA) that binds complementarily to the target DNA after PAM recognition. Once recognized, the DNA sequence is processed by two distinct nuclease domains of Cas9 that create a blunt-ended double-strand break (DBS). The HNH-like nuclease domain cleaves the target strand of the DNA substrate, while the RuvC-like nuclease domain splits the non-target strand [3].

The CRISPR/Cas9 system stands out significantly from other gene expression regulation tools due to its simplicity and compact design. However, off-target effects remain the main obstacle to its wider application [6,7,8]. Currently, most researchers apply two major approaches to reduce off-target effects: the inclusion of modifications into gRNAs [9,10,11,12] and the engineering of mutant Cas9 proteins [13,14,15,16]. The advantages of artificial RNAs lie in the simplicity of their preparation and modification.

This year, the Nobel Prize in Physiology or Medicine was awarded to K. Karikó and D. Weissman for their discoveries concerning nucleoside base modifications, which enabled the development of effective mRNA vaccines against COVID-19 [17]. They showed that the incorporation of non-canonical RNA monomers into mRNA enhances translation and can be used in therapy [18,19]. N1-methylpseudouridine (m1Ψ) is a naturally occurring RNA modification that plays an essential role in the development of mRNA-based therapeutics (Figure S1) [20]. It is on the basis of m1Ψ that two approved vaccines against COVID-19 were developed [21,22]. In addition, m1Ψ is widely used to design mRNA vaccines against other pathogens, such as HIV-1, Zika, and Ebola [23,24]. The incorporation of m1Ψ into mRNA provides a several-fold increase in protein expression in cell cultures or mice while simultaneously reducing cytotoxicity and innate immune response in vivo [25,26]. This modification contributes to many biological processes: RNA stability, translation, RNA–protein interactions, and innate immunity [20,27]. Recently, it was shown that m1Ψ can be introduced into sgRNA for further DNA substrate cleavage by CRISPR/Cas9 in vitro [28]. Given its importance in therapeutics, it is critical to explore the effect of m1Ψ in gRNAs on CRISPR/Cas9 specificity and activity.

In this study, we investigate the effect of N1-methylpseudouridine in gRNAs on the activity of the CRISPR/Cas9 system in vitro and in mammalian cells.

## 2. Results

### 2.1. Guide RNAs Modified with m1Ψ Can Cleave Plasmid DNA Substrate

To modify gRNAs, we selected N1-methylpseudouridine (m1Ψ), a naturally occurring uridine derivative. The properties of this modification have been widely studied and are currently used to increase the efficiency of therapeutic mRNAs and mRNA vaccines. Earlier, the incorporation of a m1Ψ monomer into mRNA was shown to increase its stability and translation efficiency in multiple mammalian cell lines and mouse models while also reducing cellular innate immune response [20,26,29]. In addition, two COVID-19 mRNA vaccines were developed, in which canonical uridine was replaced with m1Ψ [21]. In this study, we used plasmid and fluorescently labeled duplex DNA substrates targeting the human *ANXA6* gene. The methods were described in our previous article [30].

**Figure 1:**
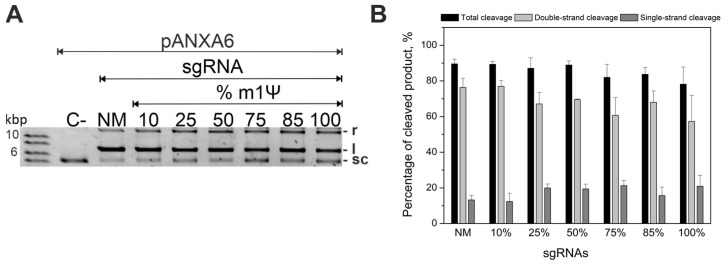
Guide RNAs with incorporated m1Ψ modification support cleavage of plasmid DNA. (**A**) Cleavage of the pANXA6 plasmid by Cas9 complexes with sgRNAs containing various numbers of m1Ψ modifications, compared to the unmodified sgRNA (NM). A plasmid without Cas9 (C−) was used as the negative control. Positive controls (C+) are presented in Figure S6. The two top bands are the cleavage products in a relaxed (r) or linear (l) form; the bottom band is the substrate (supercoiled (sc) form). The molar ratio of Cas9 RNP to target plasmid was 50:1, and the cleavage reactions were stopped after 60 min. (**B**) The percentage of plasmid cleaved by Cas9 when targeted by the modified sgRNAs. Means ± SD from three independent experiments are shown.

To achieve efficient cleavage of the plasmid using modified gRNAs and to determine a sufficient modification level, we varied the m1Ψ sgRNA modification depth from 10% to 100% during transcription in vitro (Figure 1). The percentage of cleaved plasmid did not significantly reduce with the increase of N1-methylpseudouridine substitution from 10% to 100% (Figure 1A). In particular, the percentage of cleaved plasmid for a fully modified sgRNA was comparable to that of its non-modified analog (Figure 1B).

These results indicate that sgRNA permits the entire range of m1Ψ modification depths. Furthermore, the inclusion of m1Ψ modification into gRNAs allows for the efficient cleavage of the plasmid DNA substrate in vitro.

### 2.2. Kinetics of Cas9 with Guide RNAs Containing m1Ψ Modifications

**Figure 2:**
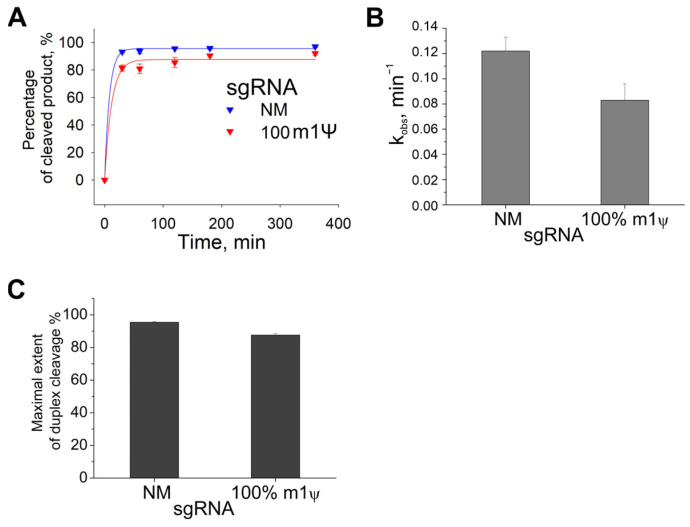
Kinetics of Cas9 with modified sgRNAs. (**A**) Kinetic curves of sgRNAs, fully modified m1Ψ, and an unmodified sgRNA (NM). (**B**) Observed rate constants (k_obs_). (**C**) The maximal extent of duplex cleavage (A_max_). Data from three independent experiments are shown as means ± s.e.m. The reactions were carried out with a 25-fold excess of Cas9/sgRNA and stopped after 30, 60, 120, 180, or 360 min.

To investigate how N1-methylpseudouridine affects the percentage of cleaved DNA duplexes in more detail, we determined the time course of DNA cleavage by Cas9 with gRNAs containing m1Ψ modifications. Using capillary gel electrophoresis and fluorescently labeled DNA duplexes (Supplementary Tables S1 and S2), we obtained the kinetic curves and calculated the observed rate constants (k_obs_) and the maximal extent of duplex cleavage (A_max_, Figure 2). The fluorescent label was located at the 5′-end of the target strand. The reaction was carried out with a 25-fold excess of Cas9/sgRNA and stopped after 30, 60, 120, 180, or 360 min.

Similar to the experiment with plasmid cleavage, a full replacement of U with m1Ψ in sgRNA reduced the percentage of cleaved product by only ~10% compared to non-modified sgRNA (NM), regardless of the reaction time (Figure 2A). A comparison of the kinetic parameters confirmed that the complete substitution of U with m1Ψ in sgRNA did not affect the reaction rate. In particular, A_max_ and k_obs_ decreased only 1.1–1.5-fold in the case of the fully modified sgRNA (Figure 2B,C).

Thus, the complexes of Cas9 and gRNAs containing m1Ψ efficiently cleave both plasmid substrate and DNA duplexes. Furthermore, m1Ψ is considered preferable to other modified base modifications for achieving a high percentage of the cleaved product.

In vitro experiments on plasmid cleavage and kinetics were also performed for modified tracrRNAs (Figure S7). It was shown that the incorporation of m1Ψ into tracrRNAs reduced the percentage of cleaved product, although high cleavage efficiency could be achieved by optimizing the reaction time and modification depth.

### 2.3. Incorporation of m1Ψ Increases CRISPR/Cas9 Specificity In Vitro

Off-target effects remain a main problem of the CRISPR/Cas9 system. Many approaches have been suggested to overcome this issue [12]. Nevertheless, only two principal strategies are used in most cases: engineering the Cas9 protein [13,14,16] and modifying gRNAs [9,12,31]. Previously, we have shown that the inclusion of non-canonical monomers, such as N6-methyladenosine, 5-methylcytidine, and pseudouridine, into gRNAs significantly increases the specificity of CRISPR/Cas9 in vitro and diminishes the immune-stimulating and cytotoxic effects of synthetic RNAs [30]. N1-methylpseudouridine in synthetic mRNAs also reduces the immune response and cytotoxicity [25]. Therefore, we decided to assess its impact on CRISPR/Cas9 specificity in vitro and generated sixteen DNA substrates, which targeted the *ANXA6* gene and contained single-nucleotide or double-nucleotide mismatches. Ten substrates (S1–S10) carried single-nucleotide mismatches in the positions 1–10 of the protospacer, and the other six (D9,10–D19,20) had double-nucleotide mismatches in the PAM-distal region of the protospacer (positions 9–20, Table S4 and Figure S8). The mutation positions were chosen considering that Cas9 tolerates multiple mismatches in the PAM-distal region and is more sensitive to them in the proximal region. In this experiment, we used sgRNAs fully modified with m1Ψ to evaluate both on- and off-target cleavage activity. The reaction was carried out with a 25-fold excess of Cas9/sgRNA and stopped after 60 min.

**Figure 3:**
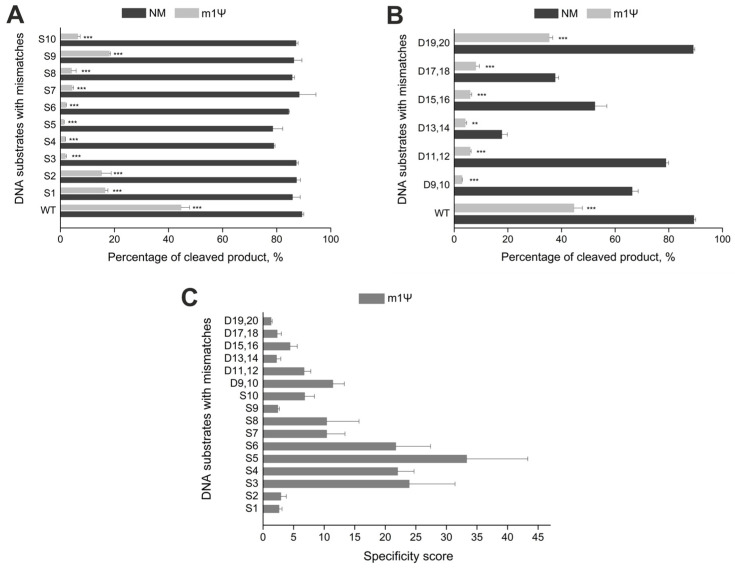
The effect of m1Ψ in gRNA on the specificity of CRISPR/Cas9 in vitro. (**A**,**B**) Percentage of cleaved product (%) for single-(**A**) and double-nucleotide mismatch-containing substrates (**B**) targeted by sgRNAs fully modified with m1Ψ or the unmodified control RNA (NM). (**C**) Specificity scores of modified sgRNA for substrates containing mismatches. The cleavage reactions of DNA duplexes were carried out with a 25-fold excess of Cas9/sgRNA and stopped after 60 min. Means ± SD from three independent experiments are shown. *p*-values are calculated using a two-tailed Student’s *t*-test (** *p* < 0.01, *** *p* < 0.001).

The non-modified sgRNA exhibited no effect on the percentage of product cleaved at off-target sites, except for the PAM-distal double-nucleotide mismatches DS13,14, DS15,16, and DS17,18 (Figure 3A,B). On the other hand, the Cas9 complex with sgRNA containing m1Ψ modification inhibited the cleavage reaction if there were single-nucleotide substitutions S3–S5 in the seed region (Figure 3A). It also significantly reduced the cleavage activity of double-nucleotide mismatches in the PAM-distal region. The only exception were the off-target sites 19–20, where virtually no differences were observed in the percentage of cleaved product between the on-target DNA and the off-target site (Figure 3B). The introduction of m1Ψ increased the specificity score for single-nucleotide and double-nucleotide mismatches (Figure 3C). In particular, the specificity score reached 20–25 for the first five single-nucleotide mismatches, peaking at 35 for the mismatch position S5. In cases of double-nucleotide mismatches, Cas9 specificity increased up to ten-fold with modified sgRNA. Thus, m1Ψ modification lead to increased specificity of CRISPR/Cas9 in vitro for all mismatch sites studied, especially those in the PAM-proximal region of sgRNA.

Comparing the results obtained with our previous data, we found that the incorporation of m1Ψ in guide RNAs allows achieving the highest specificity score than other naturally occurring modified nucleotides (Figure S9). We conclude that m1Ψ more significantly reduces off-target effects than other modifications.

### 2.4. The Effect of m1Ψ on CRISPR/Cas9 Activity in Cells

The incorporation of chemical modifications into guide RNAs has been utilized in several works to improve gene editing in human cells [10,32,33]. For example, bridged nucleic acids and locked nucleic acids can reduce off-target effects in cells [11]. Previously, we have shown that RNA base modifications in gRNAs can decrease immunogenicity and cytotoxicity [30]. Additionally, mRNAs with m1Ψ modifications are known to reduce immunogenicity in human cells [25]. Therefore, we explored the ability of sgRNAs with modified RNA nucleotides to support the Cas9-mediated gene editing in mammalian cells.

**Figure 4:**
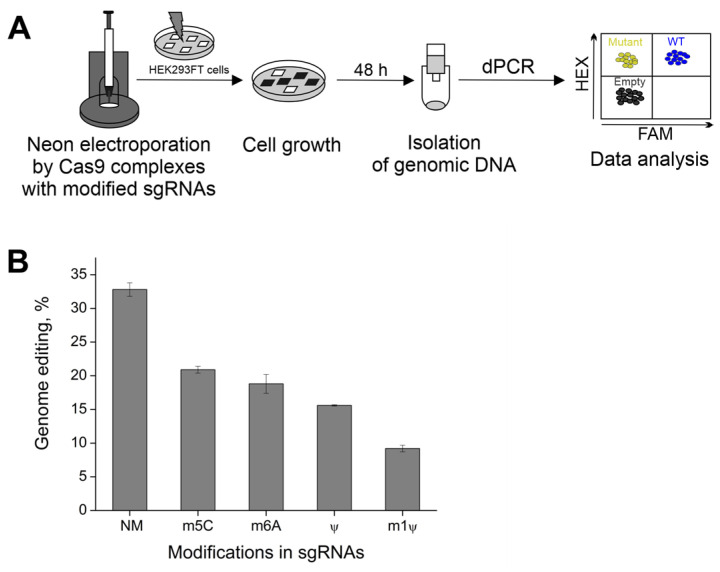
Cas9 complexes with modified sgRNAs support gene editing in human cells. (**A**) Scheme of the experiment in human cells. (**B**) The genome editing (%) for sgRNAs that were fully modified with m5C and 50% m6A, Ψ, and m1Ψ compared to the unmodified control RNA (NM). Data from three independent experiments are shown as means ± s.e.m.

To assess the effect of naturally occurring modifications on the activity of CRISPR/Cas9 in cells, we synthesized sgRNAs containing N6-methyladenosine (m6A), 5-methylcytidine (m5C), pseudouridine (Ψ), or N1-methylpseudouridine (m1Ψ). The Cas9 RNPs, assembled with modified sgRNAs, targeted the *ANXA6* gene. In this experiment, the 293FT cell line was electroporated via Cas9 NLS complexes with modified gRNAs using the Neon^TM^ Transfection System (Thermo Fisher Scientific, Waltham, MA, USA; Figure 4A). After Neon transfection, cells demonstrated 80% viability and none of the RNPs tested caused a drastic decrease in the viability rate. Genomic DNA was isolated for 48 hours post-transfection.

To evaluate the editing efficiency of RNP complexes, we utilized Qiagen digital PCR (dPCR, QIAcuity 1) and designed the primers and probes for *ANXA6.* The system of primer pair-specific probes contained a hexachlorofluorescein (HEX)-labeled reference gene probe and a 6-carboxyfluorescein (FAM)-labeled mutation-site-specific probe. The FAM-labeled probe was used to evaluate the percentage of mutant cells, and the HEX-labeled probe was used to quantify the total number of wild-type cells. Two-dimensional dPCR plots for each modified sgRNA are presented in Figure S10.

For the non-modified sgRNA, the percentage of edited cells was approximately 30% (Figure 4B). The incorporation of non-canonical nucleosides into sgRNAs slightly decreased genome editing efficiency. The editing efficiency dropped to 20% when the corresponding canonical monomers were replaced with m5C or m6A. In addition, the substitutions of U with Ψ in sgRNA approximately halved the number of mutant cells as compared to unmodified sgRNA. Still, m1Ψ modifications in gRNAs allowed for the maintenance of CRISPR/Cas9 genome editing in cells.

Taken together, these results indicate that the CRISPR/Cas9 system with modified sgRNAs can be used for genome editing in cells. The incorporation of m1Ψ modifications into gRNAs can support CRISPR/Cas9 functioning in vitro and in human cells.

## 3. Discussion

Recently, our research group succeeded in generating guide RNAs with native RNA modifications such as N6-methyladenosine (m6A), 5-methylcytidine (m5C), and pseudouridine (Ψ) [30]. We demonstrated that these modifications increase the specificity of CRISPR/Cas9 in vitro and reduce the immune-stimulating and cytotoxic effects of sgRNAs. In this study, we expanded the spectrum of naturally occurring modifications that can be incorporated into gRNAs to improve the activity of CRISPR/Cas9. We showed that Cas9 complexes with guide RNAs containing N1-methylpseudouridine enhance CRISPR/Cas9 specificity and support cleavage in vitro, including human cells.

N1-methylpseudouridine, or m1Ψ, is structurally similar to pseudouridine, Ψ. Like Ψ, m1Ψ stabilizes RNA duplexes [22,34]. However, m1Ψ leads to enhanced base stacking interactions compared to uridine or pseudouridine [20]. Previous studies have shown that the methyl group of m1Ψ increases the stability of RNA structures [35,36,37]. Our results indicate that the substitution of all U in gRNA with m1Ψ enables the cleavage of different DNA substrates in vitro, and the cleavage efficiency remains comparable to that of the unmodified gRNA analog, irrespective of the modification depth and reaction time. Furthermore, the incorporation of m1Ψ in sgRNAs significantly reduces CRISPR/Cas9 off-target effects in vitro. Among naturally occurring modifications, m1Ψ impacts Cas9 specificity the most [30].

In addition, we studied the effect of several RNA base modifications (N6-methyladenosine (m6A), 5-methylcytidine (m5C), pseudouridine (Ψ), and N1-methylpseudouridine (m1Ψ)) on the activity of CRISPR/Cas9 in cultured cells. Cas9 complexes with modified sgRNAs were observed to support genome editing in human cells, although the modifications slightly diminished the genome editing efficiency compared to non-modified sgRNA. At the same time, the incorporation of m1Ψ into sgRNA significantly decreased CRISPR/Cas9 efficiency in cells. We suppose that the observed effect of m1Ψ may result from the destabilization of the guide RNA-genome DNA complex. A recent work by Parr et al. reports that m1Ψ destabilizes RNA–DNA duplexes [38]. The destabilization occurs because m1Ψ in the RNA structure adopts the syn-conformation, preventing it from pairing with dA of DNA in the anti-conformation. According to the literature, an increase in specificity due to sgRNA modifications is accompanied by a decrease in editing efficiency [39,40]. Hence, balancing specificity and efficiency by choosing the optimal m1Ψ modification depth or combining it with other modifications can help achieve higher efficiencies in cells. Further research is required to confirm these hypotheses.

Currently, modified gRNAs allow for the improvement of many properties of the CRISPR/Cas9 system. The most studied and widely used modifications are chemical, mainly produced via solid-phase synthesis [12]. In contrast, the effect of naturally occurring RNA modifications on CRISPR/Cas9 activity has been poorly studied. Among this type of modification, 2′-*O*-methylated ribonucleotides have been extensively explored [41]. Previous studies have shown that incorporating three 2′-O-Me groups or combining them with other modifications at the 5′- and 3′-terminus of sgRNAs can enhance both the chemical stability and target specificity of sgRNAs [40]. In addition, 2′-O-Me modifications increase gene editing efficiency in mammalian cells [32,39]. Only recently has the effect of other native RNA base modifications, such as N1-methyladenosine, N6-methyladenosine 2-thiouridine, and 4-thiouridine, been investigated in vitro and in HEK293T cells [42]. No direct correlation between the stabilization effect of modification and CRISPR/Cas9 activity has been observed, and m6A was found to be the most potent modification.

In the context of therapeutic applications, it is crucial to address CRISPR/Cas9 challenges, such as low efficiency of gene editing, its off-target effects, and high cytotoxicity. Earlier, it was demonstrated that the incorporation of m1Ψ into mRNAs increases mRNA stability and reduces immunogenicity in vivo [25]. Thus, gRNAs containing m1Ψ are likely to be more stable, and these modifications can reduce immune-stimulating and cytotoxic effects. Another advantage of m1Ψ over alternative chemical modifications is the simplicity and cost-effectiveness of modified RNAs, as T7 transcription in vitro enables the production of fully modified sgRNAs with high yield in a short time.

## 4. Materials and Methods

### 4.1. Synthesis of Guide RNAs In Vitro

Short crRNA was chemically synthesized on the automatic synthesizer ASM800 (Biosset, Novosibirsk, Russia) using the standard solid-phase phosphitamide method. All tracrRNAs and sgRNAs (Table 1) were obtained via T7 in vitro transcription, with DNA templates synthesized via PCR from the pSpCas9(BB)-2A-GFP plasmid (PX458, Addgene #48138, Teddington, UK, Figure S2).

The transcription of tracrRNA and sgRNA was executed in vitro at 37 °C for 4 h. To obtain fully modified tracrRNAs/sgRNAs, a modified NTP, m1ΨTP (Biolabmix, Novosibirsk, Russia), was used instead of the UTP monomer. The depth of modification was varied by changing the m1ΨTP/UTP ratio. The sgRNAs containing N6-methyladenosine (m6A), 5-methylcytidine (m5C), or pseudouridine (Ψ) were synthesized using the same approach as the m1Ψ-modified gRNAs. The transcription was carried out following our protocol previously described in [30].

### 4.2. Preparation of the Cas9 Protein

For in vitro experiments, the pMJ806 plasmid that encodes *S. pyogenes* Cas9 carrying an N-terminal His6 tag, maltose-binding protein sequence, and TEV protease cleavage site was obtained from Addgene (#39312, Figure S3). For transfection, the pMJ915 plasmid encoding *S. pyogenes* Cas9 with two C-terminal SV40 NLS and tags similar to the plasmid described above was obtained from Addgene (#69090, Figure S4). Both Cas9 proteins were overexpressed and purified according to the previously described protocol [43].

### 4.3. Biochemical In Vitro DNA Plasmid Cleavage Assays

In vitro cleavage assays were performed as previously described [30]. The pANXA6 plasmid carrying the *ANXA6* cDNA insert (pANXA6, OriGene # RC202086, Rockville, ML, USA, Figure S5) was used in the assays. Before the experiment, crRNA was hybridized with tracrRNA (0.2 μM each) in water at 95 °C for 30 s followed by 3 min on ice. If sgRNA was used, this step was omitted. The Cas9 enzyme and gRNA were combined (final concentrations: 0.2 μM Cas9, 0.2 μM tracrRNA, and 0.2 μM crRNA, or 0.2 μM Cas9 and 0.2 μM sgRNA) in the reaction buffer (20 mM HEPES pH 7.5, 100 mM KCl, 5% glycerol, 1 mM DTT, 0.5 mM EDTA, 2 mM MgCl_2_). The plasmid substrate was added to a 50-fold excess of Cas9/gRNA (sgRNA or crRNA:tracrRNA) in the reaction buffer. The reactions (10 µL) were allowed to proceed at 37 °C for 1 h and then quenched by adding 10 µL proteinase K (60 mM EDTA, 4 M urea, 0.4 mg/mL proteinase K) and incubating at 37 °C for 15 min. The products were resolved via electrophoresis in 1% agarose gel, stained with 0.5 µg/mL ethidium bromide, and visualized via a UV imager.

### 4.4. In Vitro DNA Duplex Cleavage

Fluorescently labeled DNA duplexes (Table S1) were generated via PCR amplification of plasmids with fluorescently labeled primers (Table S2). The fluorescently labeled DNA substrates encoded a fragment of *ANXA6*, and the target substrate strands contained a fluorescein label at the 5′-end. The experiments were performed under the same conditions as described above, except that the reactions were carried out with a 25-fold excess of Cas9/gRNA and stopped after 60 min. The products of the fluorescent substrate cleavage were analyzed via capillary electrophoresis on the ABI 3500 Genetic Analyzer (Thermofisher, Waltham, MA, USA).

### 4.5. Cell Culture

The human embryonic kidney cell line 293FT was maintained at 37 °C with 5% CO_2_ incubation in a 1:1 mixture of Dulbecco’s Modified Eagle’s Medium and Ham’s F12 Medium (DMEM/F12, Gibco, Waltham, MA, USA) supplemented with 10% fetal bovine serum (FBS), 1% MEM NEAA, sodium pyruvate, GlutaMax, and antibiotic–antimycotic (all Gibco, USA).

### 4.6. Neon Transfection of 293FT Cells

We prepared a 10-μL mixture of sgRNA and spCas9 protein (100 pmol each) in Resuspension Buffer (R buffer, included in Neon Kits) and kept it at room temperature (20–25 °C) for 15 min to form the Cas9 RNP complex. 293FT cells were harvested, washed, and divided into separate Eppendorf tubes at 2 × 10^5^ cells per tube. Cells were resuspended in R Buffer with Cas9 RNPs. Transfection was performed according to the manufacturer’s protocol using a 10-μL tip with the following parameters: 1450 V/30 ms/1 pulse. 293FT cells resuspended in R buffer with no additives and subjected to the manufacturer’s standard “negative” transfection (0 V/1 ms/0 pulses) were used as a negative control. After transfection, cells were transferred into 500 μL DMEM/F12 supplemented media with 10% FBS and no antibiotics and incubated at 37 °C in a humidified CO_2_ incubator. Two days after transfection, genomic DNA (gDNA) was extracted using the D-cells-250 kit (Biolabmix, Novosibirsk, Russia). The editing efficiency (%) was analyzed using digital PCR.

### 4.7. Digital PCR

We performed digital PCR (dPCR) assays to estimate the editing efficiency on the *ANXA6* target gene. Sequence-specific PCR primers and probes were designed using Primer3 plus (http://primer3plus.com (accessed on 2 September 2023) by Eurofins (Eurofins Genomics, Toronto, ON, Canada). Primer and probe sequences are listed in Table 2.

The reference gene probe was 5′-HEX-labeled, and the mutation-site-specific probe was 5′-FAM-labeled. Both types of probes were quenched with BHQ1 at the 3′ end. The dPCR was performed on the QIAcuity One Platform System (Qiagen, Hilden, Germany). The final reaction mixture volume was 40 µL:10 µL of 4× QIAcuity Probe PCR Master Mix (Qiagen, 250102), 15 ng of gDNA, primers (0.8 µM), reference probe (0.4 µM), mutant probe (0.04 µM), and HindIII restriction enzyme (0.25 U/μL, New England BioLab, Hitchin, UK). Each reaction was prepared in a pre-plate and then transferred into the 24-well QIAcuity Nanoplate (Qiagen, 250001). The thermocycling protocol was as follows: (1) 95 °C—2 min, (2) 95 °C—15 s and 60 °C—30 s for 40 cycles. The final imaging step included the reading of both mutant (FAM channel) and reference (HEX channel) signals. Data was analyzed with the QIAcuity Suite Software V1.1.3 193. Gene editing efficiency was calculated as the ratio of mutant partitions (only HEX-positive droplets) to wild-type partitions (HEX/FAM-double positive droplets) [44].

### 4.8. Statistics and Quantification

All calculations and formulas are presented in our previous article [30]. The images of gel electrophoresis were quantified using Quantity One 4.6.8 (Bio-Rad Laboratories, Hercules, CA, USA). The percentages of single- and double-strand breaks of plasmid (SSB and DSB, respectively) were calculated as:

$$SSB,\%=\frac{V_{r}}{V_{r}+V_{l}+V_{sc}\cdot k}\times 100\%$$

$$DSB,\%=\frac{V_{l}}{V_{r}+V_{l}+V_{sc}\cdot k}\times 100\%$$

Total cleavage, % = Double strand cleavage + Single strand cleavage

where V is the intensity of bands corresponding to the relaxed (V_r_), linear (V_l_) and supercoiled (V_sc_) plasmid, and k = 1.14 is the ethidium bromide intercalation coefficient in the supercoiled plasmid.

The kinetic parameters were calculated in the SigmaPlot 11.0 program. Statistical analyses were performed using a two-tailed Student’s *t*-test. Differences were considered significant at *p* < 0.05. *p*-values are shown in the figures and Table S3.

## 5. Conclusions

In summary, we show that the incorporation of N1-methylpseudouridine into guide RNAs preserves on-target genome editing while significantly reducing CRISPR-Cas9 off-target effects in vitro. In addition, this naturally occurring modification supports CRISPR/Cas9 activity in human cells. Given the properties of N1-methylpseudouridine leveraged in mRNA therapy, it may become a valuable tool for genome editing systems.

## Figures and Tables

**Table 1:** Sequences of RNAs used in the experiments.

Name	Structure (5′–3′)
crRNA	atgcagctaatacgactcactataggtcagggttactatgataagg
tracrRNA	ucuagcaaguuaaaauaaggcuaguccguuaucaacuugaaaaaguggcaccgagucggugcuuuuuu
sgRNA	gguuggacaugcucgacauucguuuuagagcuagaaauagcaaguuaaaauaaggcuaguccguuaucaacuugaaaaaguggcaccgagucggugcuuuuuu

**Table 2:** Amplification primers and probes for digital PCR.

Name	Structure (5′-3′)
F _ANX_dPCR	cctctcaagtccatgttcccc
R _ANX_dPCR	ggaatgtccaagagactccca
Probe_cleav_FAM_ANX	[FAM]acattcgggagatcttccggaccaagt[BHQ1]
Probe_ref_HEX _ANX	[HEX]agctcatgggggcagagaagggagca[BHQ1]

## Data Availability

All data generated or analyzed during this study are included in this article.

## Supplementary Materials

The supporting information can be downloaded at: https://www.mdpi.com/article/10.3390/ijms242317116/s1.
