# Supplementary material for: Influence of N1-Methylpseudouridine in Guide RNAs on CRISPR/Cas9 Activity
Source: Int J Mol Sci. 2023 Dec 4;24(23):17116. doi: 10.3390/ijms242317116 (PMC10707292; doi:10.3390/ijms242317116)
Supplement: Supplementary file 1 [file ijms-24-17116-s001.zip › Supplementary Tables_revision.pdf]

Supplementary Tables

Table S1. Sequence of target strand of DNA duplexes used as substrates for Cas9/sgRNA or crRNA:tracrRNA cleavage.

Name	Structure (5'-3')
DNA_target strand<br>(531nt)	TTGTGTCTTCGTCAGTCCCGAGTCCCTTCATGGCTTTCCGC<br>AGCGCTTTGGCATCTGCGTCAGGGTTGAAGTCATTGGCTG<br>GGCGCACAGTTCCCTTCAGCTCTACTCGGGCCACTGCACT<br>AAGTTCCCACATCTGATAGGCCACCTGCGCTGCCTCCGGG<br>AAGAACTGGCCAGCAGCATCATCATCTCCCCCAGACAGCT<br>TCAGCAGAGTCTTCTTGTACTCGCCAGAGGTGTCATTCTT<br>GATCATGCTGTAGAGGGACTTCTCATACTTGGTCCGGAAG<br>ATCT**CCC**GAATGTCGAGCATGTCCAACTCACTACGGGAG<br>ACCATGATGCGGATCAGGGTGTTGTCCCGAGTCCCCAGGC<br>CCTTCATAGCCTTGAAGAGCCTTTCAGCAAAATATTCCGG<br>GGTGCTCCGGATACACTTCACTACGGCCAGCATTAGCTTC<br>TCAAAGTCCCCAGACAGCTCCCCTCGGATGCTGGCTTCAA<br>TCGGCTTCCCTGTGGTCTTCAGATACTCATCGAACACCAA<br>CCGAAGATGC

The spacer in the DNA duplex is underlined and the PAM is in red and bold.

Table S2. Amplification primers used to generate the fluorescently labeled DNA duplexes.

Name	Structure (5'-3')
F _ANX	gcatcttcggttggtgttcg
R-FAM_ANX_target strand	FAM-ttgtgtcttcgtcagtcccg
F_ANX_mismatch	aatattttgctgaaaggctcttcaa

Table S3. Calculated P-values for the specificity experiment (for Figure 3A, B).

Name of sample	P-value
sgRNA_ m1Ψ_WT	0,00058
sgRNA_ m1Ψ_S1	0,00014
sgRNA_ m1Ψ_S2	0,00053
sgRNA_ m1Ψ_S3	0,00003
sgRNA_ m1Ψ_S4	0,00005
sgRNA_ m1Ψ_S5	0,00042
sgRNA_ m1Ψ_S6	0,00000
sgRNA_ m1Ψ_S7	0,00056
sgRNA_ m1Ψ_S8	0,00004
sgRNA_ m1Ψ_S9	0,00036
sgRNA_ m1Ψ_S10	0,00001

sgRNA_m1Ψ_D9,10	0,00011
sgRNA_m1Ψ_D11,12	0,00007
sgRNA_ m1Ψ_D13,14	0,00146
sgRNA_ m1Ψ_D15,16	0,00055
sgRNA_ m1Ψ_D17,18	0,00063
sgRNA_ m1Ψ_D19,20	0,00020

Table S4. Sequences of mutant DNA substrates used for the experiments determining specificity of the CRISPR-Cas9 system.

**Name; S- single-point mismatch; D- double-point mismatches**	**Sequence, spacer region (5'-3')**
WT	GAATGTCGAGCATGTCCAAC
S1	CAATGTCGAGCATGTCCAAC
S2	GTATGTCGAGCATGTCCAAC
S3	GATTGTCGAGCATGTCCAAC
S4	GAAAGTCGAGCATGTCCAAC
S5	GAATCTCGAGCATGTCCAAC
S6	GAATGACGAGCATGTCCAAC
S7	GAATGTGGAGCATGTCCAAC
S8	GAATGTCCAGCATGTCCAAC
S9	GAATGTCGTGCATGTCCAAC
S10	GAATGTCGACCATGTCCAAC
D9,10	GAATGTCGTCCATGTCCAAC
D11,12	GAATGTCGAGGTTGTCCAAC
D13,14	GAATGTCGAGCAACTCCAAC
D15,16	GAATGTCGAGCATGAGCAAC
D17,18	GAATGTCGAGCATGTCGTAC
D19,20	GAATGTCGAGCATGTCCATG

Mismatches in DNA duplexes are underlined.
