# Supplementary material for: Influence of N1-Methylpseudouridine in Guide RNAs on CRISPR/Cas9 Activity
Source: Int J Mol Sci. 2023 Dec 4;24(23):17116. doi: 10.3390/ijms242317116 (PMC10707292; doi:10.3390/ijms242317116)
Supplement: Supplementary file 1 [file ijms-24-17116-s001.zip › Supplementary_full_revision.pdf]

Supplementary Materials

Table S1. Sequence of target strand of DNA duplexes used as substrates for Cas9/sgRNA or crRNA:tracrRNA cleavage.

Name	Structure (5′-3′)
DNA_target strand (531nt)	TTGTGTCTTCGTCAGTCCCGAGTCCCTTCATGGCTTTCCGCAGCGCT TTGGCATCTGCGTCAGGGTTGAAGTCATTGGCTGGGCGCACAGTTC CCTTCAGCTCTACTCGGGCCACTGCACTAAGTTCCCACATCTGATA GGCCACCTGCGCTGCCTCCGGGAAGAACTGGCCAGCAGCATCATC ATCTCCCCCAGACAGCTTCAGCAGAGTCTTCTTGTACTCGCCAGAG GTGTCATTCTTGATCATGCTGTAGAGGGACTTCTCATACTTGGTCC GGAAGATCT**CCC**GAATGTCGAGCATGTCCAACTCACTACGGGAGA CCATGATGCGGATCAGGGTGTTGTCCCGAGTCCCCAGGCCCTTCAT AGCCTTGAAGAGCCTTTCAGCAAAATATTCCGGGGTGCTCCGGAT ACACTTCACTACGGCCAGCATTAGCTTCTCAAAGTCCCCAGACAG CTCCCCTCGGATGCTGGCTTCAATCGGCTTCCCTGTGGTCTTCAGAT ACTCATCGAACACCAACCGAAGATGC

The spacer in the DNA duplex is underlined and the PAM is in red and bold.

Table S2. Amplification primers used to generate the fluorescently labeled DNA duplexes.

Name	Structure (5′-3′)
F _ANX	gcatcttcggttggtgttcg
R-FAM_ANX_target strand	FAM-ttgtgtcttcgtcagtcccg
F_ANX_mismatch	aatattttgctgaaaggctcttcaa

Table S3. Calculated P-values for the specificity experiment (for Figure 3A, B).

Name of sample	P-value
sgRNA_ m1Ψ_WT	0,00058
sgRNA_ m1Ψ_S1	0,00014
sgRNA_ m1Ψ_S2	0,00053
sgRNA_ m1Ψ_S3	0,00003
sgRNA_ m1Ψ_S4	0,00005
sgRNA_ m1Ψ_S5	0,00042
sgRNA_ m1Ψ_S6	0,00000
sgRNA_ m1Ψ_S7	0,00056
sgRNA_ m1Ψ_S8	0,00004
sgRNA_ m1Ψ_S9	0,00036
sgRNA_ m1Ψ_S10	0,00001
sgRNA_m1Ψ_D9,10	0,00011

sgRNA_m1Ψ_D11,12	0,00007
sgRNA_ m1Ψ_D13,14	0,00146
sgRNA_ m1Ψ_D15,16	0,00055
sgRNA_ m1Ψ_D17,18	0,00063
sgRNA_ m1Ψ_D19,20	0,00020

Table S4. Sequences of mutant DNA substrates used for the experiments determining specificity of the CRISPR-Cas9 system.

**Name; S- single-point mismatch; D- double-point mismatches**	**Sequence, spacer region (5′-3′)**
WT	GAATGTCGAGCATGTCCAAC
S1	CAATGTCGAGCATGTCCAAC
S2	GTATGTCGAGCATGTCCAAC
S3	GATTGTCGAGCATGTCCAAC
S4	GAAAGTCGAGCATGTCCAAC
S5	GAATCTCGAGCATGTCCAAC
S6	GAATGACGAGCATGTCCAAC
S7	GAATGTGGAGCATGTCCAAC
S8	GAATGTCCAGCATGTCCAAC
S9	GAATGTCGTGCATGTCCAAC
S10	GAATGTCGACCATGTCCAAC
D9,10	GAATGTCGTCCATGTCCAAC
D11,12	GAATGTCGAGGTTGTCCAAC
D13,14	GAATGTCGAGCAACTCCAAC
D15,16	GAATGTCGAGCATGAGCAAC
D17,18	GAATGTCGAGCATGTCGTAC
D19,20	GAATGTCGAGCATGTCCATG

Mismatches in DNA duplexes are underlined.

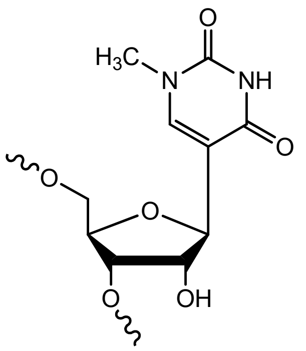

Figure S1. Structure of N1-methylpseudouridine (m1Ψ).

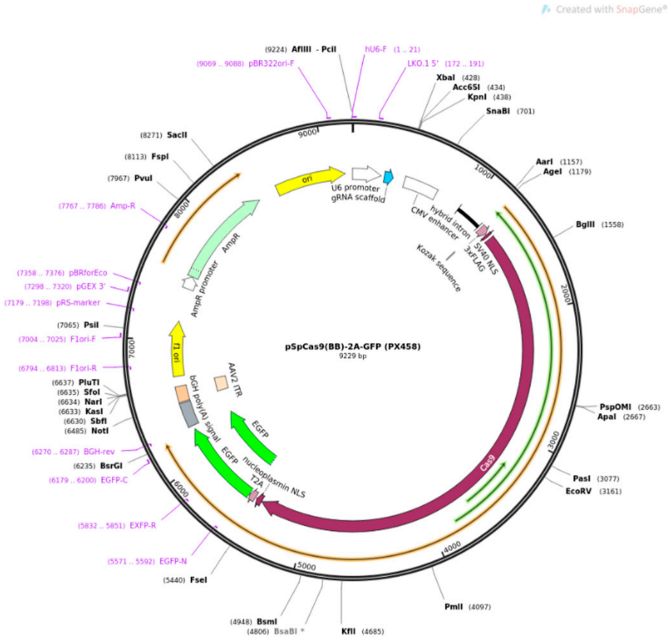

Figure S2. Map of PX458 plasmid

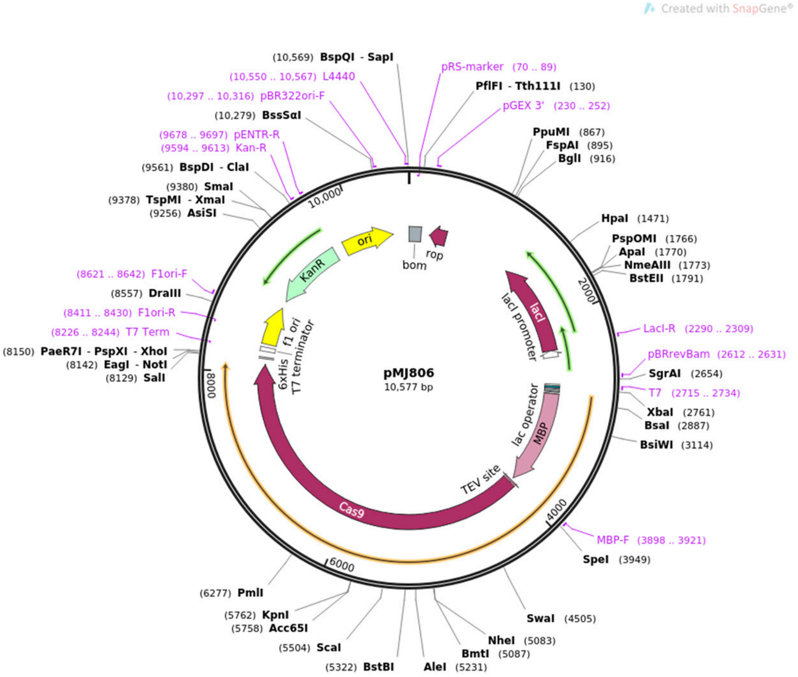

Figure S3. Map of pMJ806 plasmid

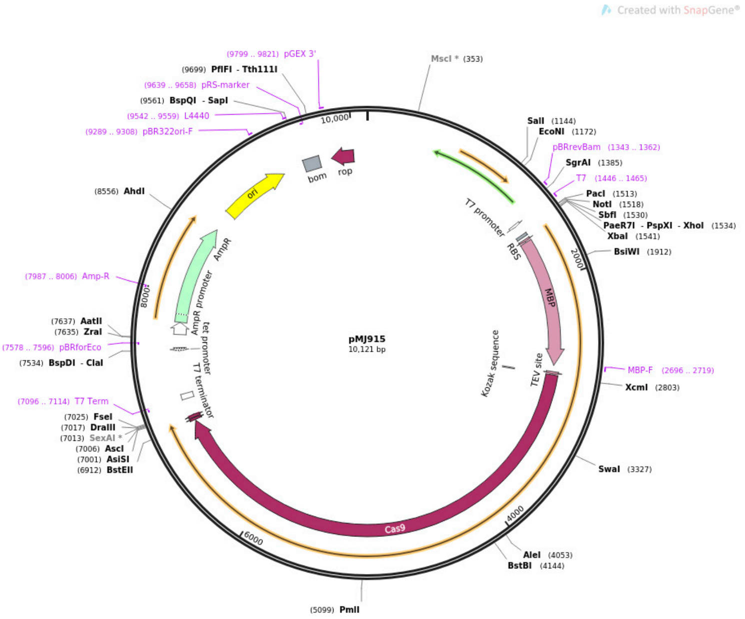

Figure S4. Map of pMJ915 plasmid

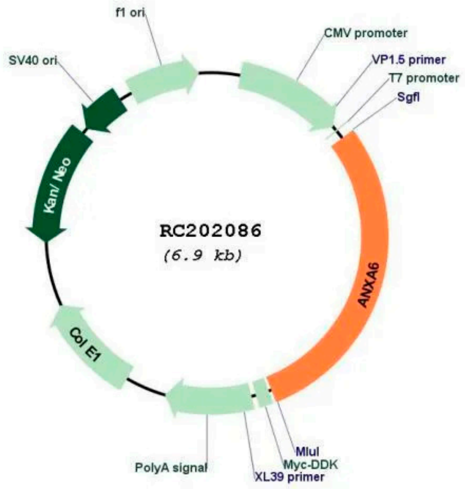

Figure S5. Map of pANXA6 plasmid

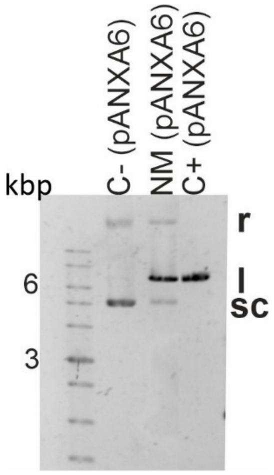

Figure S6. The controls of plasmids in the cleavage assay. Plasmids pANXA6 without Cas9 (C-) were used as negative controls. Cleavage of plasmids by Cas9 complex with unmodified sgRNA. To obtain positive controls (C+), the plasmids were digested with restriction enzymes: MIuI (pANXA6). Linear (l) and supercoiled (sc) forms are indicated

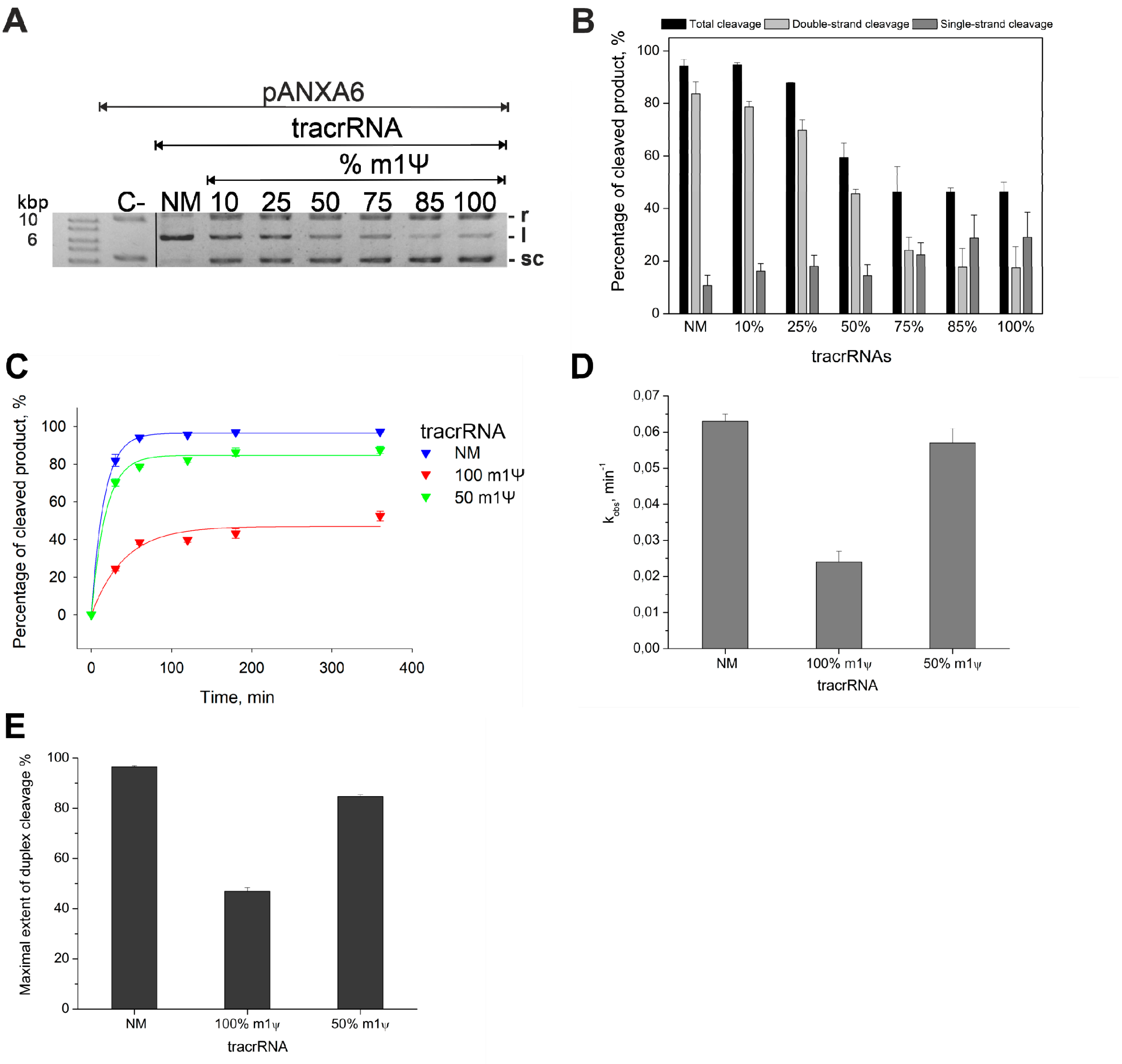

Figure S7. The effect of N1-methylpseudouridine in tracrRNA on the efficiency of the CRISPR/Cas9 system *in vitro*. (**A**) Cleavage of the pANXA6 plasmid by Cas9 with crRNA and tracrRNAs, modified to various m1Ψ depth, and with unmodified tracrRNA (NM). Plasmid without Cas9 (C-) was used as negative control. The two top bands are the cleavage products (relaxed (r) and linear (l) form), while the bottom band is the substrate (supercoiled (sc) form). The molar ratio of Cas9 RNP to target plasmid was 50:1 and the cleavage reactions were stopped after 60 min. (B) The percentage of cleaved plasmid by Cas9 targeted by the modified tracrRNAs. (C) Time-course of duplex cleavage by Cas9/crRNA:tracrRNA with m1Ψ modification 100 and 50%. Unmodified tracrRNA (NM) was used as reaction control. (D) Observed

rate constants (k_{obs}). (E) Maximal extent of duplex cleavage (A_{max}). Data are shown as means ± SD from three independent experiments.

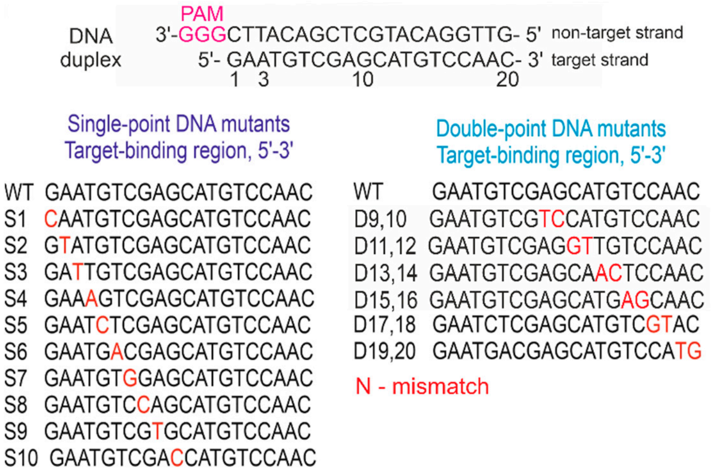

Figure S8. The protospacer sequences of DNA substrates with single-point (S1-S10) or double-point (D9,10-D19,20) mismatches. A set of sixteen DNA substrates with mismatches related to the Psp1 protospacer from the ANXA6 gene The Psp1 sequences of DNA substrates with single- (S1-S10) or double-nucleotide (D9,10-D19,20) mismatches, marked in orange. The DNA numbers of the substrates correspond to the positions of the mutations from the 5′-end. WT, control unmodified DNA duplex.

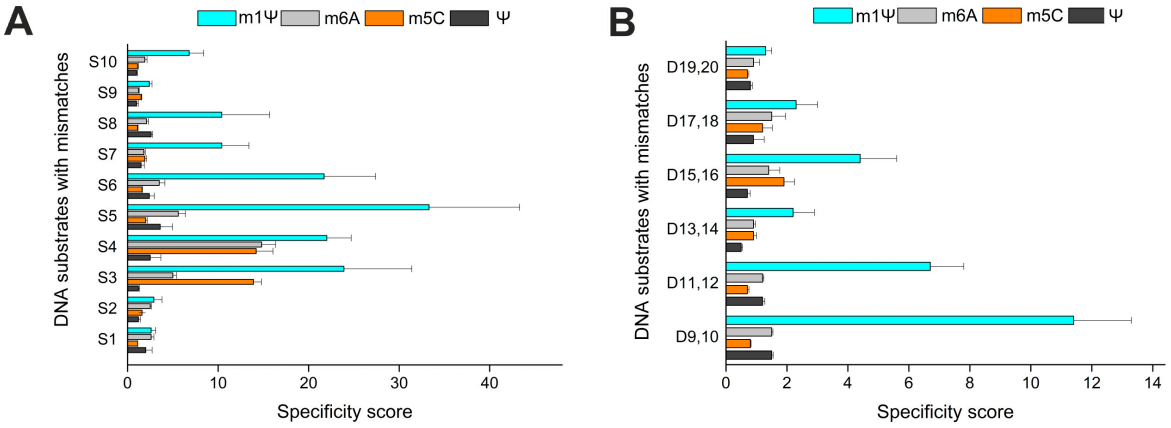

Figure S9. The effect of natural occurring modifications in gRNA on the specificity of CRISPR/Cas9 in vitro. (A, B) Specificity scores for single- (**A**) and double-nucleotide mismatch-containing substrates (**B**) targeted by sgRNAs modified with m1Ψ, m6A, m5C and Ψ. The cleavage reactions of DNA duplexes were carried out with a 25-fold excess of Cas9/sgRNA and stopped after 60 min. Means ± SD from three independent experiments are shown.

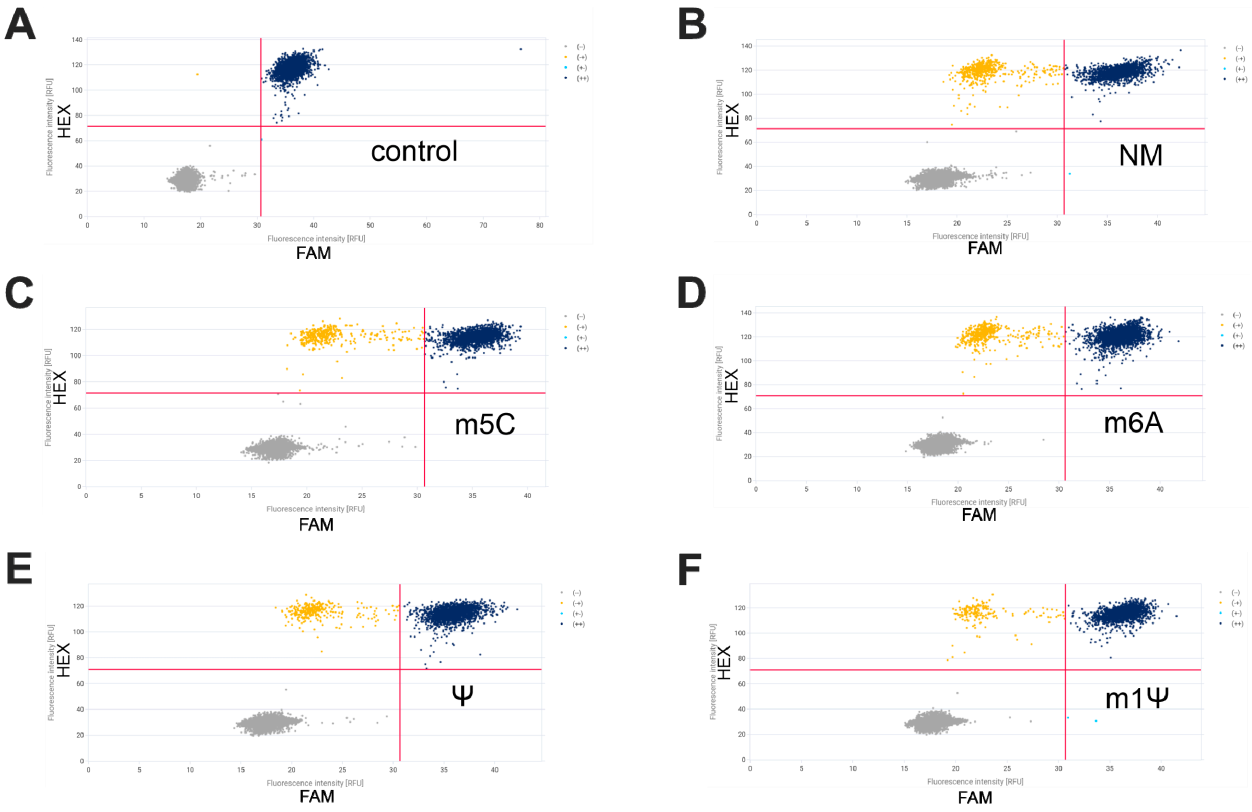

Figure S10. Two-dimensional dPCR plots for modified sgRNAs. (A-F) The results of dPCR for HEK293T control cells (A), for unmodified control RNA NM (B) and for sgRNAs modified with 5-methylcytidine-m5C (C) and 50% N6-methyladenosine-m6A (D), pseudouridine -Ψ (E) and N1-methylpseudouridine m1Ψ (F). The Y axis shows fluorescence intensity for the HEX channel (reference probe) and the X axis shows the fluorescence intensity for the FAM channel (mutation-site-specific probe).
