# Supplementary figures and images for: Influence of N1-Methylpseudouridine in Guide RNAs on CRISPR/Cas9 Activity
Source: Int J Mol Sci. 2023 Dec 4;24(23):17116. doi: 10.3390/ijms242317116 (PMC10707292; doi:10.3390/ijms242317116)

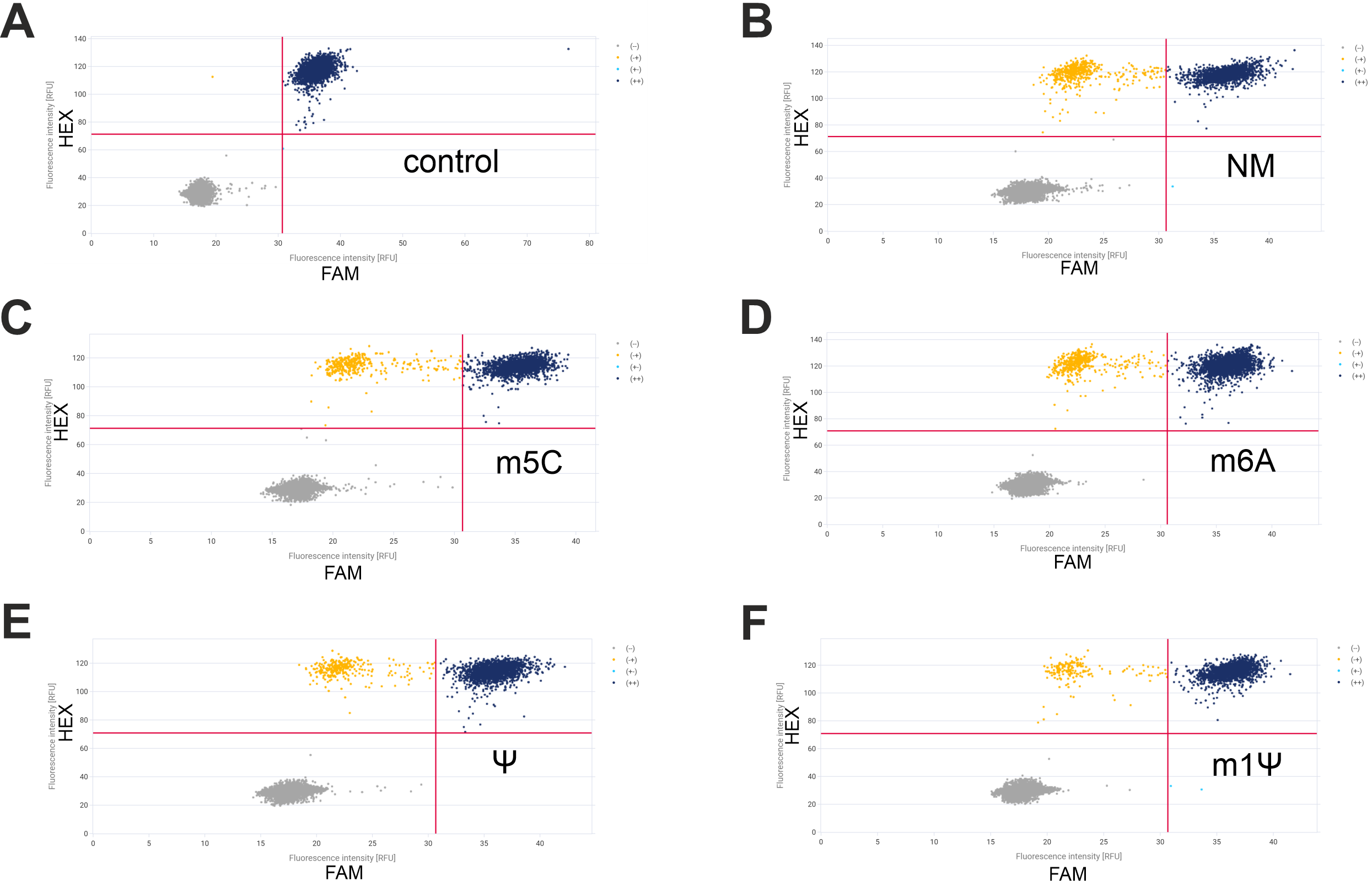

Supplement: Supplementary file 1 [file ijms-24-17116-s001.zip › Figure S10.tif]

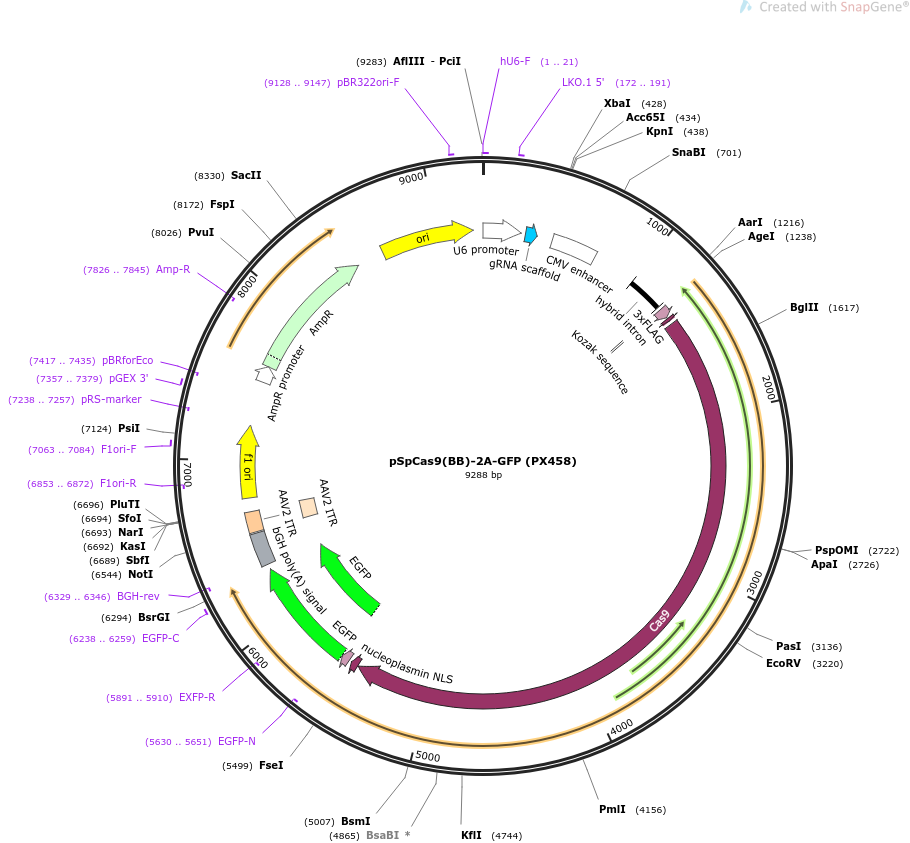

Supplement: Supplementary file 1 [file ijms-24-17116-s001.zip › Figure S2.png]

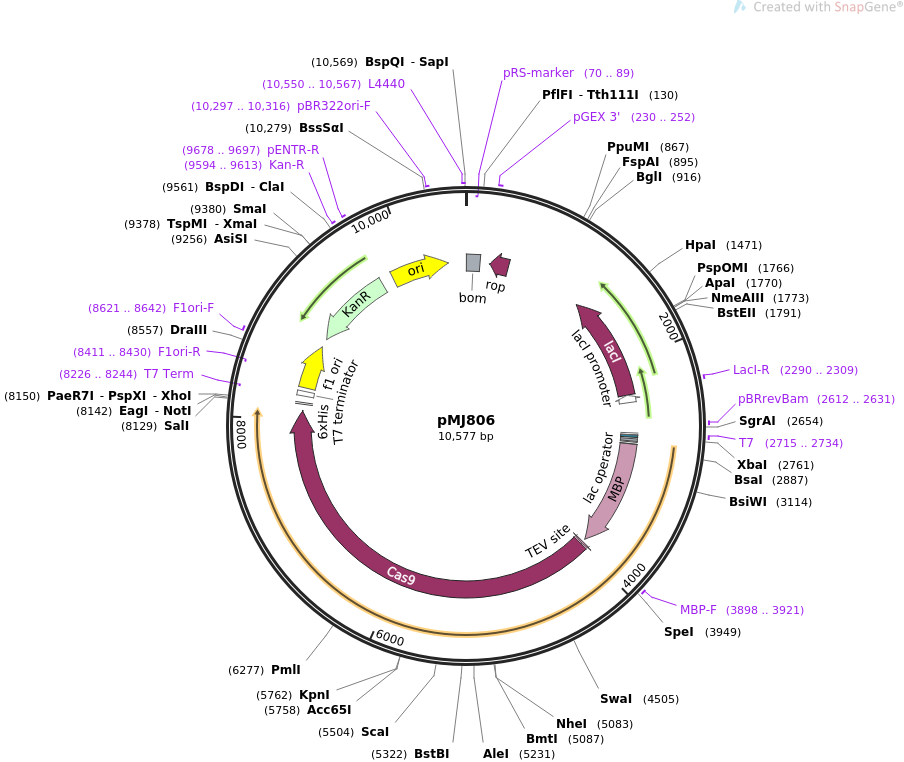

Supplement: Supplementary file 1 [file ijms-24-17116-s001.zip › Figure S3.png]

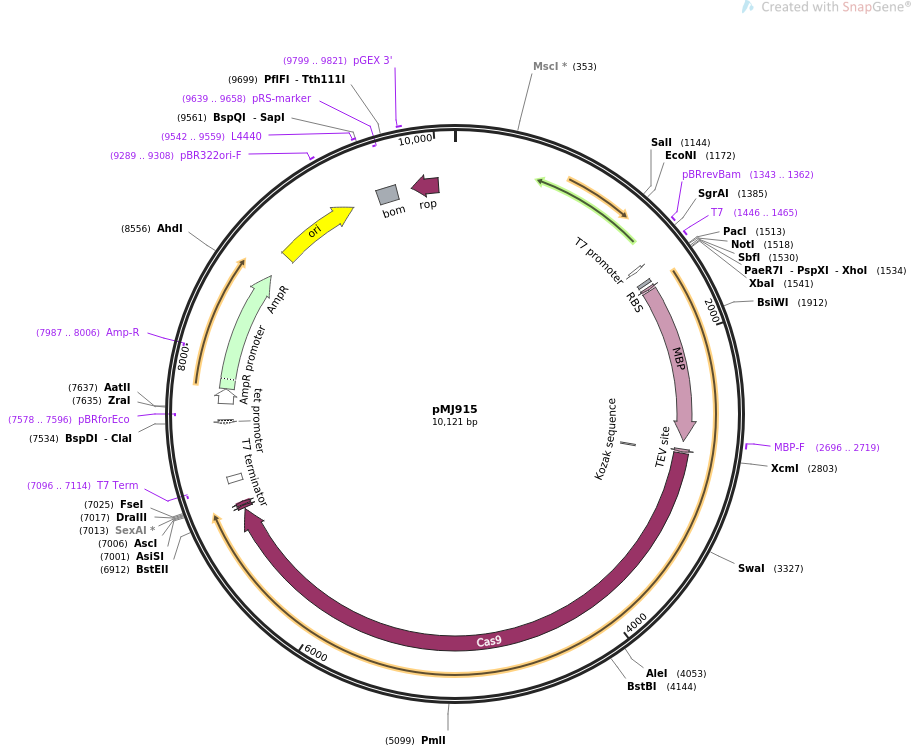

Supplement: Supplementary file 1 [file ijms-24-17116-s001.zip › Figure S4.png]

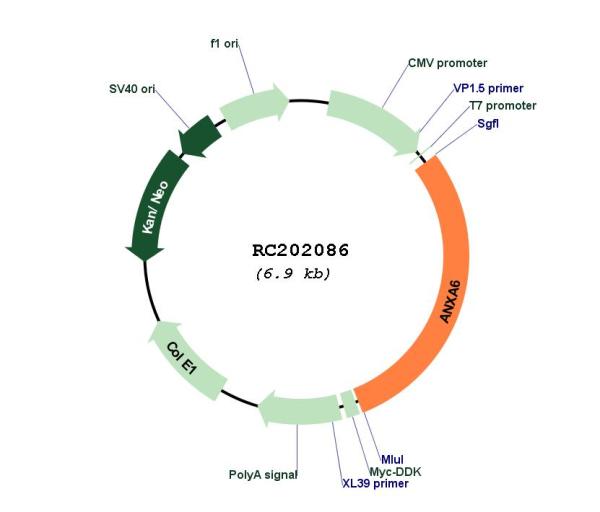

Supplement: Supplementary file 1 [file ijms-24-17116-s001.zip › Figure S5.jpg]

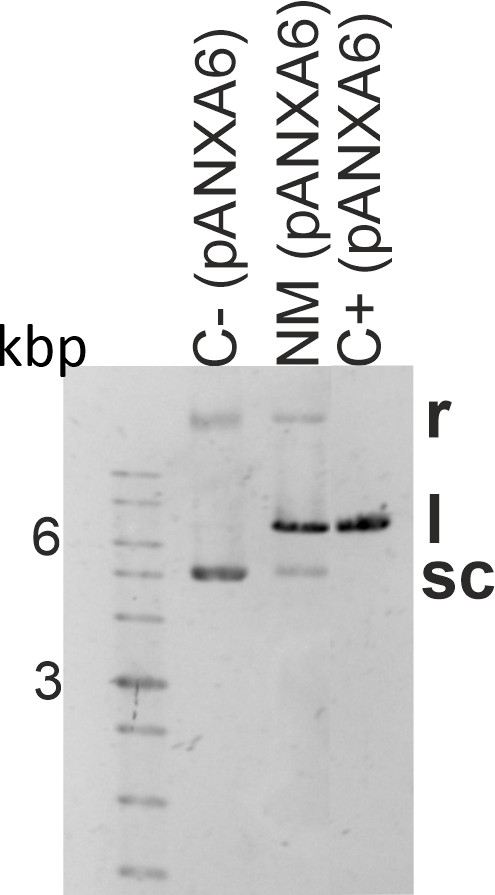

Supplement: Supplementary file 1 [file ijms-24-17116-s001.zip › Figure S6.tif]

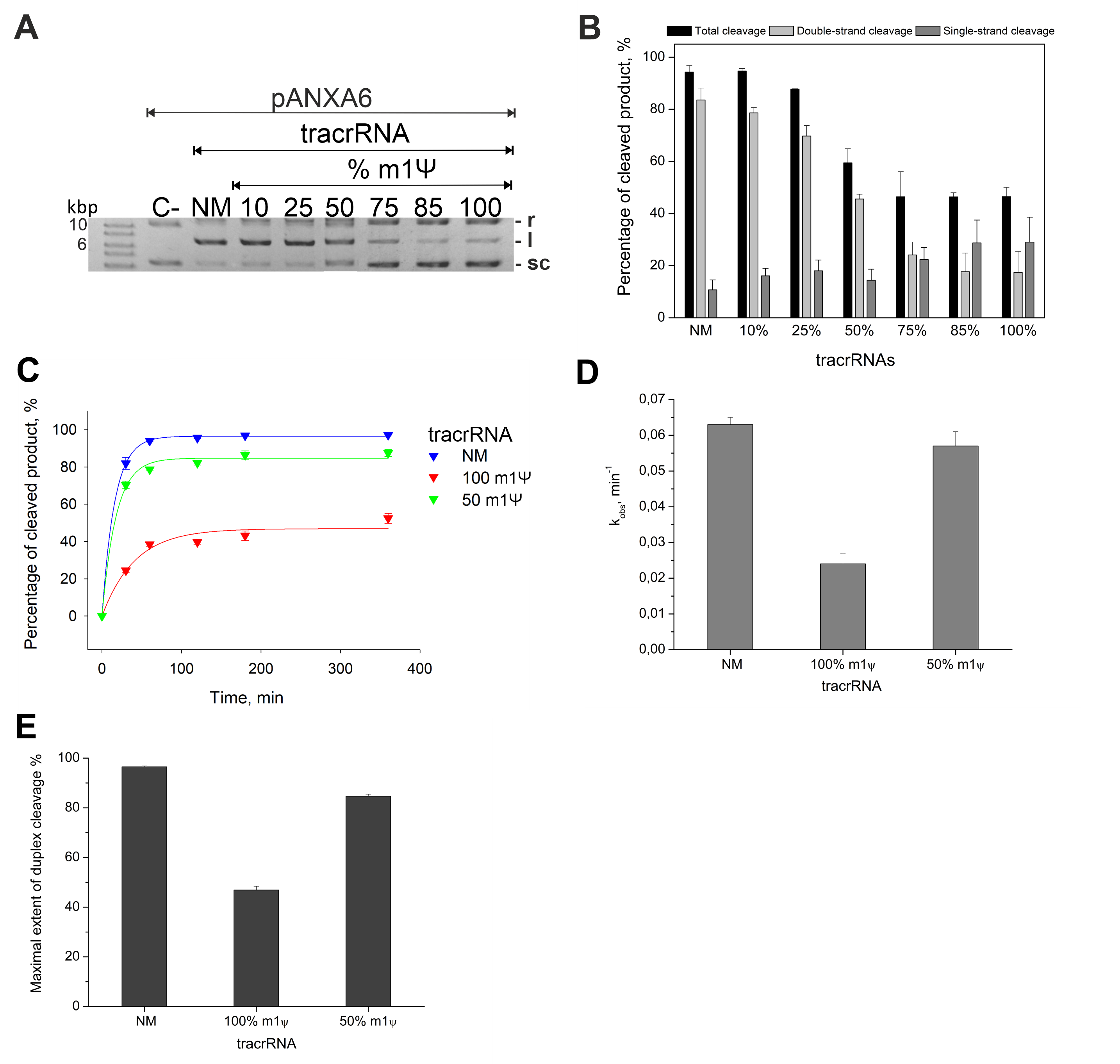

Supplement: Supplementary file 1 [file ijms-24-17116-s001.zip › Figure S7.tif]

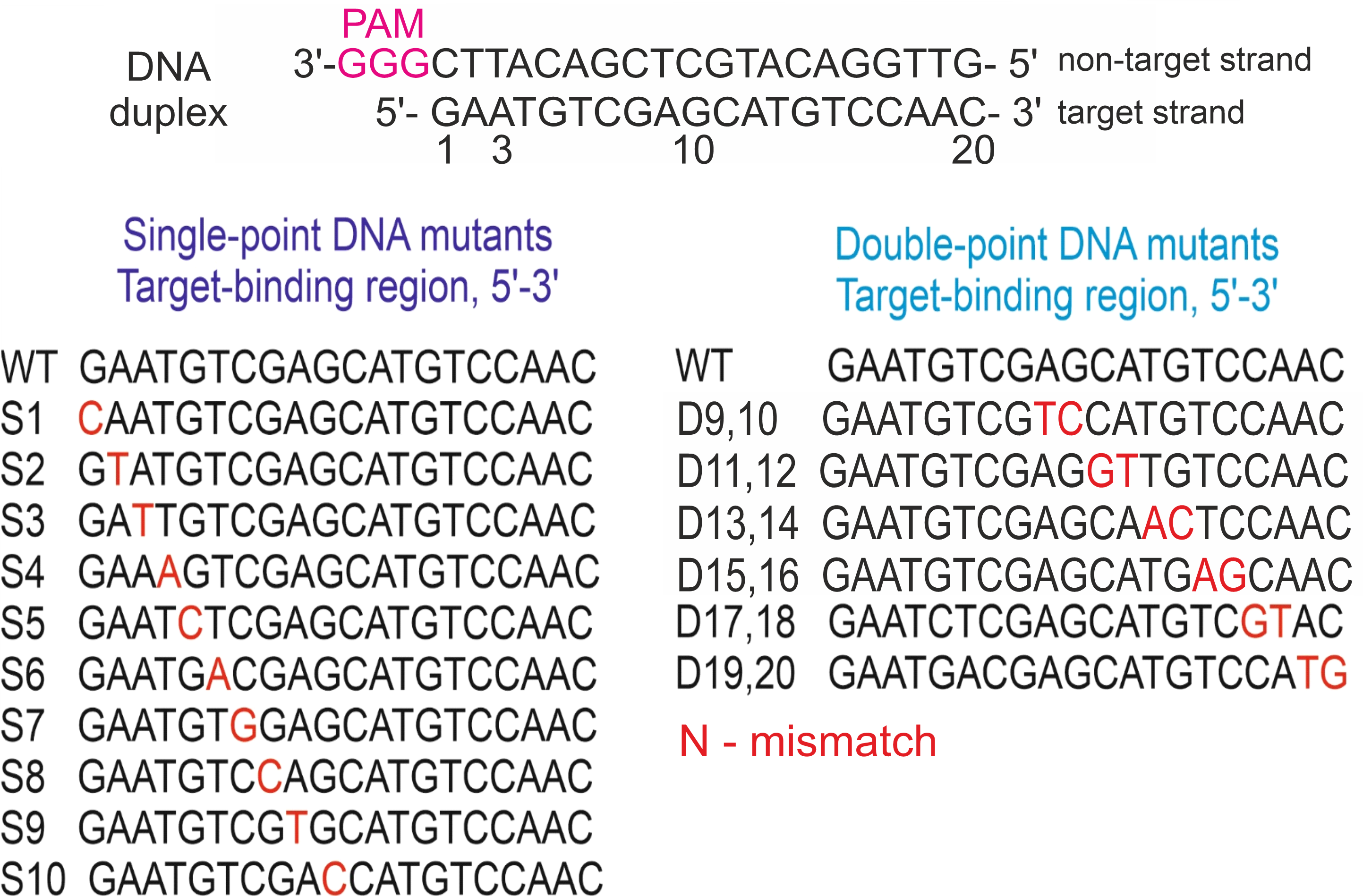

Supplement: Supplementary file 1 [file ijms-24-17116-s001.zip › Figure S8.tif]

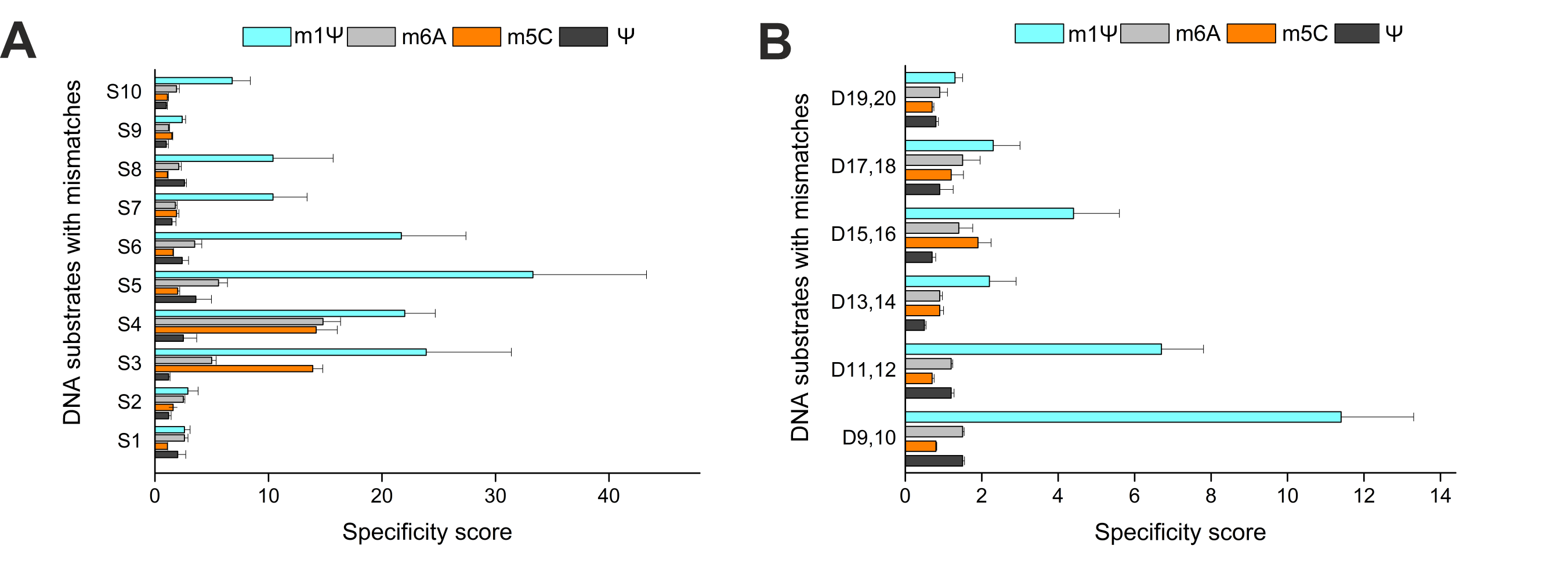

Supplement: Supplementary file 1 [file ijms-24-17116-s001.zip › Figure S9.tif]
